# The Role of Extracellular Vesicles in Liver Fibrosis: Friends or Foes?

**DOI:** 10.3390/biomedicines12122665

**Published:** 2024-11-22

**Authors:** Xiang Tao, Can Chen, Mei Liu

**Affiliations:** 1 Department of Gastroenterology, Tongji Hospital, Tongji Medical College, Huazhong University of Science and Technology, Wuhan 430030, China; 2 Clinical Center of Human Gene Research, Union Hospital, Tongji Medical College, Huazhong University of Science and Technology, Wuhan 430022, China

**Keywords:** extracellular vesicles, liver fibrosis, migrasomes, exosomes, intracellular communication, therapeutic strategy

## Abstract

Liver fibrosis represents a common pathway in the progression of various chronic liver diseases towards cirrhosis and liver failure. Extracellular vesicles (EVs) are membrane-enclosed particles secreted by diverse cell types, including exosomes, microvesicles, apoptotic vesicles, and the recently identified migrasomes. These vesicles can be taken up by recipient cells, thereby modulating their function through the transport of cargo molecules. EVs facilitate intercellular communication and play a significant role in the development of liver fibrosis. Moreover, the detection of EVs in various body fluids offers sensitive diagnostic tools for assessing liver fibrosis. Additionally, EVs may serve as therapeutic targets, potential therapeutic agents, and drug delivery vehicles. This article reviews recent advances in the field of EVs concerning liver fibrosis and related diseases, with a particular focus on the potential role of the newly discovered migrasomes in intracellular crosstalk within the liver.

## 1. Introduction

The liver is one of the largest internal organs in the human body and performs a variety of important metabolic, detoxification, synthetic, and immune functions [1]. It is also a highly heterogeneous organ that contain multiple cell types, such as hepatocytes, stellate cells, Kupffer cells (KCs), endothelial cells, and bile duct epithelial cells (cholangiocytes) [2]. All these different types of liver cells can secrete extracellular vesicles (EVs), which play an important role in liver fibrosis [3]. Progressive liver fibrosis, caused by chronic viral and metabolic disorders, results in over one million deaths from cirrhosis each year. Despite this, there are currently no approved anti-fibrotic treatments available [4]. Fibrotic liver disease manifests as excessive accumulation of extracellular matrix (ECM), angiogenesis, and impaired immune balance, ultimately leading to the progression of liver cirrhosis and hepatocellular carcinoma (HCC) [5]. Further research on EVs has the potential to contribute to the diagnosis and treatment of liver fibrosis. EVs can be classified into exosomes, microvesicles, and apoptotic bodies [6]. Recently, migrasomes, a newly discovered type of EVs generated during cell migration, have become a promising tool for cell communication [7]. In this review, we summarize the classification, biogenesis, mediation of cellular communication, and therapeutic roles of EVs in liver fibrosis.

## 2. Classification, Biogenesis, and Isolation of EVs

### 2.1. Classification and Biogenesis of EVs

**Figure 1:**
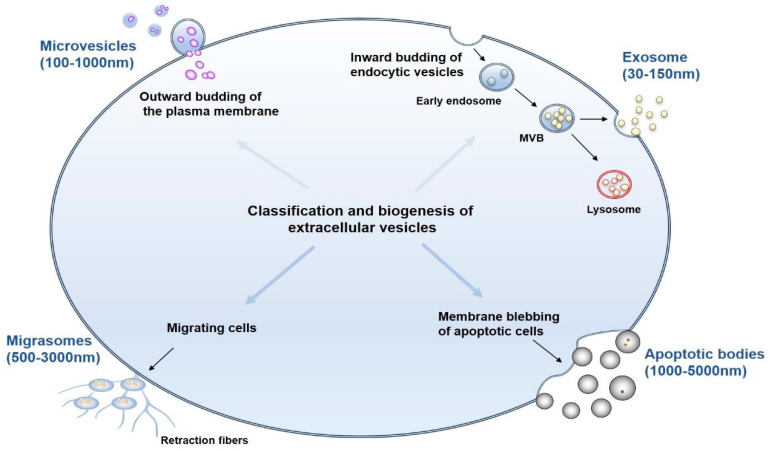
The classification and biogenesis of extracellular vesicles. EVs are membrane-enclosed particles secreted into the extracellular space by a variety of cells. They can be classified into four main subtypes: exosomes, microvesicles, apoptotic bodies and migrasomes. MVB, multivesicular body.

EVs are membrane-enclosed particles secreted into the extracellular space by a variety of cells, carrying various nucleic acids, proteins, lipids, and metabolites [8,9]. Based on their biogenesis, release pathway, size, content, and function, they can be categorized into four major subtypes: exosomes, microvesicles (MVs), apoptotic bodies, and migrasomes [10]. Among them, exosomes are derived from inward budding of endocytic vesicles that mature into multivesicular bodies (MVBs) and are released by fusion of MVBs with the plasma membrane in the size range of 30 to 150 nm. MVs are generated and released by outward budding of the plasma membrane and range in size from 100 to 1000 nm. Apoptotic bodies are formed by membrane blebbing of apoptotic cells and contain cellular debris in the size range of 1000 to 5000 nm [11,12,13]. Migrasomes are a recently discovered type of EVs that are typically generated along retraction fibers in migrating cells, most of which are elliptical with a diameter of 500–3000 nm. They are oval-shaped vesicles formed during cell migration and contain proteins, RNAs, organelles, and numerous smaller vesicles. Under scanning electron microscopy, they exhibit a resemblance to pomegranate-like structures [14,15,16] (Figure 1).

The formation of migrasomes is governed by a tightly regulated process modulated by several key molecules, including sphingomyelin synthase 2 (SMS2), integrins, phosphatidylinositol-4-phosphate 5-kinase type I α (PIP5K1α), and tetraspanins [17]. Among these, the assembly of SMS2 occurs at the leading edge of migrating cells, a process that initiates the formation of migrasomes [18]. Tetraspanins are a family of cell-surface glycoproteins consisting of 33 members, found in all cell types, each containing four transmembrane domains [19]. Tetraspanin 4 has been identified as a major component of the migrasome membrane [16]. A recent study suggested that migrasome formation involves the assembly of cholesterol- and tetraspanin-rich membrane microdomains into large micrometer-sized domains, then swelling to form migrasomes. This study finds that both tetraubiquitin 4 and cholesterol are critical for the formation of migrasomes [20]. Additionally, recent findings indicate that calcium ions may enhance migrasome formation through synaptotagmin-1 [21]. Despite these advancements, the molecular mechanisms underlying migrasome formation remain largely unexplored.

### 2.2. Isolation of EVs

Ultracentrifugation is often regarded as the “gold standard” for nanoparticle extraction and has been extensively employed to isolate EVs from various sources. One such technique, differential ultracentrifugation, involves gradually increasing the centrifugal speed to separate samples of differing sizes. However, it has certain limitations, such as lower purity, yield, and integrity, and longer processing time. Additionally, ultrafiltration provides a rapid and selective means of separation through the microporous structure of a semi-permeable membrane, making it suitable for large-scale production. The third method, size exclusion chromatography, relies on the molecular size of the components being analyzed and is effective for separating EVs of uniform size [22]. Finally, ExoQuick polymer precipitation utilizes a polymer (e.g., PEG) to modify the solubility and dispersion of EVs, prompting their aggregation and subsequent precipitation from solution, which is then achieved through centrifugation. This method is characterized by a short separation time, straightforward execution, and relative gentleness on EVs [23]. Moreover, it has been shown that EVs can be separated efficiently by combining these methods.

In addition to conventional separation techniques, microfluidic separation techniques are becoming better known, including four types: active, passive, immunity-based, and combined. Studies have been performed to isolate exosomes from whole blood by integrating two techniques, like the integration of acoustic and microfluidic techniques [23]. Recently, exosome detection via the ultrafast-isolation system (EXODUS) has been identified as an ultra-fast separation system for the isolation of exosomes with high purity and yield [24].

Under normal physiological and pathological conditions, EVs are important mediators of intercellular communication [25]. They can interact with or enter different cell types through their carriers and influence the function and fate of recipient cells. Recently, EVs have also been investigated as biomarkers, therapeutic targets, or potential therapeutic agents and carriers for drug delivery in different diseases [26].

## 3. The Role of EVs (Mainly Exosomes and MVs) in Liver Fibrosis

Liver diseases account for 4% of all deaths worldwide (1 out of every 25 deaths) [27]. They are a diverse group of diseases involving multiple factors and mechanisms. Liver fibrosis represents a major healthcare burden worldwide caused by multiple chronic liver injury, ultimately leading to cirrhosis and its complications, portal hypertension, liver failure, and hepatocellular carcinoma (HCC) [28,29,30]. The hallmark of liver fibrosis is activation of hepatic stellate cells (HSCs), which are the major cellular source of matrix-producing myofibroblasts. Currently, effective treatments for liver fibrosis and cirrhosis are very limited, relying mainly on liver transplantation for decompensated cirrhotic liver disease [31]. The development and progression of these liver diseases are related to EVs [9]. EVs can transfer signaling molecules between different types of liver and non-liver cells and regulate processes such as metabolism, immunity, fibrosis, and carcinoma. Moreover, EVs can also serve as biomarkers of liver disease, reflecting the level of liver function and damage [32].

### 3.1. EVs as Critical Biomarkers or Mediators in Liver Fibrosis

Recent evidence has underscored the significance of EVs as critical biomarkers or mediators in liver fibrosis and cirrhosis [33]. Circulating EV-contained microRNAs (miRs), specifically miR-192-5p, miR-194-5p, miR-22-3p, and miR-29a-3p, along with hepatic injury RNA markers, have been identified as indicators of severe cholestasis-induced liver fibrosis [34]. Cholangiocyte-derived exosomal long non-coding RNA (lncRNA) H19 has been demonstrated to promote cholestatic liver fibrosis by regulating HSC differentiation and activation, thus representing a promising diagnostic biomarker for this condition [35]. Additionally, circulating let-7 levels in plasma and EVs correlate with the progression of hepatic fibrosis in chronic hepatitis C [36]. Furthermore, EVs can be isolated from the blood of patients with cirrhosis, reflecting the pathophysiological status and prognosis of these individuals. For instance, levels of hepatocyte MVs significantly enhance the prediction of six-month mortality in patients with cirrhosis [37]. Mechanically, circulating EVs have been reported to induce functional impairment in circulating monocytes, contributing to sepsis and high mortality rates in cirrhosis [38]. Similarly, EVs derived from endothelial cells are indicative of severe hepatopulmonary syndrome condition in cirrhosis [39]. Notably, promising proteins found in the EVs of patients’ serum, including thrombospondin-1, fibulin-1, and fibrinogen gamma chain, can differentiate healthy controls from individuals with liver cancer or cirrhosis [40]. In another study, serum EVs from patients diagnosed with metabolic dysfunction-associated steatotic liver disease (MASLD) were analyzed for liver fibrosis through biopsy, revealing that the concentration of serum fibulin-3 was significantly higher in patients with advanced fibrosis [41]. Therefore, EVs demonstrate considerable translational value in the diagnosis of liver fibrosis, and further efforts are required to integrate these findings into clinical practice.

### 3.2. The Role of EVs in the Progression of Liver Fibrosis

Numerous studies have underscored the critical role of EVs derived from hepatocytes in the progression of liver fibrosis. For instance, research conducted by Liu et al. demonstrated that β-arrestin 1 enhances the release of mannan-binding lectin serine protease 1 (MASP1)-enriched small EVs from hepatocytes, which subsequently activates HSCs and promotes liver fibrosis [42]. Similarly, hepatocyte-derived exosomal H2AFJ has been shown to facilitate HSC migration and invasion during liver fibrosis through the MAPK/STMN1 signaling pathway [43]. Moreover, EVs from fat-laden hepatocytes modulate the phenotype of HSCs [44,45]. Specifically, miR-128-3p-enriched EVs derived from lipotoxic hepatocytes are internalized by HSCs, leading to HSC activation via PPAR-γ targeting [46]. Additionally, lipotoxic hepatocyte-derived LIMA1-enriched small EVs play a crucial role in promoting HSCs activation in non-alcoholic fatty liver disease (NAFLD)-related liver fibrosis by negatively regulating PINK1-mediated mitophagy. Another study indicated that free fatty acid (FFA) treatment induces the formation and secretion of EVs, promoting the release of ferritin from hepatocytes through these vesicles. Subsequently, ferritin within the EVs is taken up by HSCs, which contributes to the accelerated activation of these cells. Conversely, the depletion of ferritin cargo from EVs has been shown to restore liver function and mitigate fibrosis associated with NAFLD [47]. In addition, exosomes released from damaged hepatocytes have been found to activate TLR3 in HSCs, thereby exacerbating liver fibrosis by enhancing IL-17A production from γδ T cells [48]. In cultured mouse primary hepatocytes, exposure to acetaminophen or carbon tetrachloride (CCl_4_) led to mitochondrial dysfunction, which resulted in the release of mitochondrial DNA (mtDNA) from damaged hepatocytes to neighboring HSCs and hepatocytes via EVs. This process facilitated fibrotic responses in activated HSCs and hepatocyte injury [49]. Another study has shown that EVs can be secreted from hepatocytes in response to exposure to aflatoxin B1, thereby promoting HSC activation and hepatocytotoxicity and subsequent liver fibrosis [50].

Beyond hepatocytes, HSCs have also been reported to release platelet-derived growth factor receptor alpha (PDGFRα) and glycolysis-related protein-enriched EVs that promote hepatic fibrosis [51,52]. EVs derived from PDGFRα-overexpressing cells have been shown to enhance HSC migration in vitro and contribute to liver fibrosis in vivo. Inhibition of PDGFRα enrichment in serum EVs alleviated liver fibrosis [51]. Additionally, glycolysis in HSCs is elevated during liver fibrosis, leading to increased expression of the EV-related protein RAB31, which further enhances EV release and promotes the expression of fibrosis-related genes in recipient HSCs, thereby amplifying liver fibrosis [53]. Moreover, activated HSCs can release exosomes that mediate glycolysis in liver nonparenchymal cells by delivering glycolysis-associated proteins, including pyruvate kinase M2 (PKM2) and glucose transporter 1 (GLUT1) [52].

**Figure 2:**
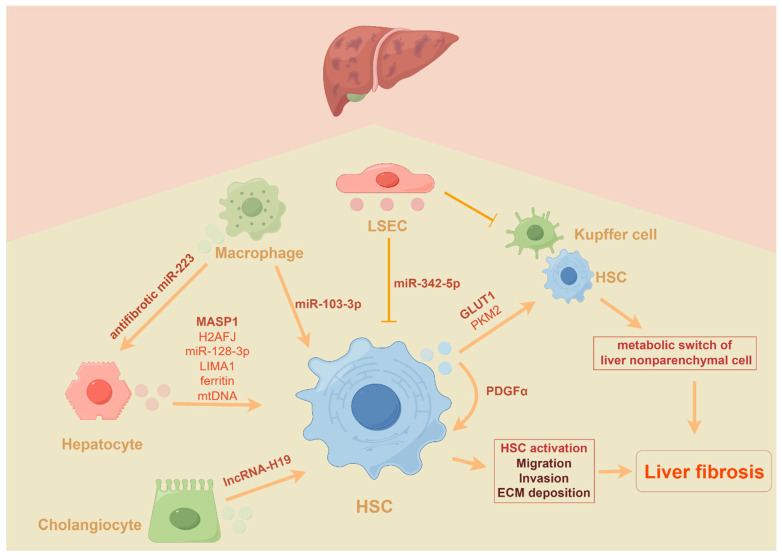
EVs are critical players in the progression of liver fibrosis by transferring signaling molecules between different types of liver and non-liver cells. The cartoon highlights different cells (hepatocytes, cholangiocytes, macrophages, endothelial cells, etc.) can secrete EVs enriched with a variety of molecules (mtDNA, miR-128-3p, lncRNA-H19, miR-103-3p, etc.) to regulate the activity of hepatic stellate cells and thus influence the progression of liver fibrosis. ECM, extracellular matrix; HSCs, hepatic stellate cells; KCs, Kupffer cells; lncRNA, long non-coding RNA; LSECs, liver sinusoidal endothelial cells; miR, microRNA; mtDNA, mitochondrial DNA.

Recent studies have demonstrated that myeloid-specific interleukin-6 (IL-6) signaling inhibits NAFLD-associated liver fibrosis by delivering exosomes enriched with anti-fibrotic miR-223 to hepatocytes [54]. Notably, cholangiocyte-derived exosomal lncRNA H19 has been shown to contribute to the progression of cholestatic liver fibrosis by promoting the differentiation and activation of HSCs [35]. In response to lipopolysaccharide (LPS) treatment, exosomes containing miR-103-3p released from THP-1 macrophages may significantly influence the crosstalk between macrophages and HSCs during the progression of liver fibrosis [55]. Under physiological conditions, liver sinusoidal endothelial cells (LSECs) transmit inhibitory signals to HSCs through the secretion of miR-342-5p-enriched EVs. However, in pathological conditions, elevated serum aldosterone levels have been reported to induce autophagic degradation of MVBs in LSECs, resulting in a decrease in both the quantity and quality of EVs, thereby activating HSCs and promoting the development of liver fibrosis [56]. Additionally, another study has confirmed that LSEC-derived EVs may attenuate the fibrogenic phenotype of activated HSCs and the inflammatory phenotype of KCs [57]. Overall, the current evidence positions EVs as critical players in the progression of liver fibrosis and underscores the necessity for further investigation into biomarkers within EVs derived from various cell types (Figure 2).

### 3.3. The Application of EVs (Mainly Exosomes and MVs) in Liver Fibrosis

EVs can be used as therapeutic targets or agents for liver fibrosis, achieving anti-fibrotic effects by inhibiting stellate cell activation and reducing inflammation and apoptosis, among other mechanisms. A study by Li et al. has demonstrated the protective effects of tetramethylpyrazine against liver fibrosis by targeting hepatocyte-derived and mtDNA-enriched EVs [49]. Another study showed that fibronectin type III domain-containing protein 5 (FNDC5)/irisin could attenuate liver fibrosis via inhibiting the release of HSC-derived exosomes [58].

Notably, serum EVs from healthy individuals possess inherent anti-fibrogenic and anti-fibrotic properties, containing microRNAs that exert therapeutic effects on activated HSCs or injured hepatocytes [59]. Currently, EVs from various sources have shown good therapeutic effects in liver fibrosis by delivering different cargos to target pathways in HSCs or macrophages. For instance, exosomes derived from three-dimensional human embryonic stem cells (3D-hESC) have been shown to inhibit liver fibrosis by delivering the anti-fibrotic miR-6676-3p to activated HSCs [60]. Mesenchymal stem cells (MSCs) represent another source of exosomes that can induce ferroptosis in HSCs by transferring BECN1 and suppress the viability and migration of HSCs through the transfer of miR-618, thereby alleviating fibrosis in mouse liver models [61,62]. Additionally, circDIDO1, another cargo in MSC-derived EVs, has been shown to inhibit activation of HSCs by targeting miR-141-3p [63]. Exosomes derived from human bone MSCs (hBM-MSCs) have also been found to suppress HSC activation via the Wnt/β-catenin pathway [64]. Recently, placenta MSC-derived EVs containing miR-378c have been demonstrated to inhibit HSC activation by suppressing epithelial–mesenchymal transition (EMT) [65]. Adipose-derived mesenchymal stem cells (ADSCs) produce miR-150-5p-enriched EVs that reduce the proliferation and activation of HSCs by targeting CXCL1 [66]. Furthermore, exosomes derived from ADSCs may inhibit the progression of liver fibrosis by regulating choline metabolism through the PI3K/Akt/mTOR pathway [67]. In addition to regulating HSCs, amnion-derived MSC-EVs have been shown to inhibit the activation of KCs in rat models [68]. Another study indicated that MSC-derived EVs may also modulate the phenotype of macrophages by delivering miR-148a to them [69]. Moreover, exosomes derived from human umbilical cord-MSCs (hucMSCs) have been reported to inhibit EMT and protect hepatocytes, thereby attenuating liver fibrosis [70]. A recent study showed that the small EVs of MSCs accumulated mainly in macrophages located in damaged areas of the liver. In addition, the therapeutic effect of small EVs was not necessarily dose dependent, and it reached a plateau when the dosage exceeded a certain level [71].

Additionally, EVs derived from human-induced pluripotent stem cells (iPSCs) have demonstrated significant therapeutic potential in a murine model of liver fibrosis, with miR-92a-3p identified as the most abundant miRNA [72]. Liver stem cells (LSCs) represent a pluripotent population of adult stem cells in the liver that exhibit a mesenchymal-like phenotype. These cells secrete EVs that play a crucial protective role in liver fibrosis by delivering anti-fibrotic miRNAs, such as miR-146a-5p [73]. In murine models of NASH, LSC-derived EVs demonstrate both anti-fibrotic and anti-inflammatory effects, which attenuate the progression of liver fibrosis [74]. Recently, another study identified miR-142-5p in LSC-EVs, which displays a protective role in liver fibrosis by regulating macrophage polarization via CTSB [75]. Collectively, stem cell-derived EVs may represent an effective treatment for liver fibrosis.

In addition to various sources of stem cells, a growing body of research suggests that mesenchymal stromal cells play a crucial role in the treatment of liver fibrosis. Tonsil-derived mesenchymal stromal cells (T-MSCs) represent a novel source of small EVs that may inhibit HSC activation by suppressing hedgehog signaling through miR-486-5p [76]. Adipose-derived stromal cells (ASCs) demonstrate limited efficacy in treating liver fibrosis, primarily influenced by graft efficiency and immune response. However, exosomes derived from ASCs exhibit enhanced anti-fibrotic effects, primarily through their impact on HSC activation and the reprogramming of hepatocyte glutamate metabolism [77]. Human umbilical cord perivascular cells (HUCPVCs)-derived EVs possess anti-fibrotic properties by overexpressing insulin-like growth factor 1 (IGF-1) using adenoviral vectors [78]. Furthermore, exosomes from umbilical cord blood (UCB) plasma may inactivate HSCs by inhibiting TGF-β/ID-1 signaling, which leads to an improvement in liver fibrosis [79]. Human-induced hepatocytes (hiHep) can be expanded in vitro, making their cell culture supernatant a nearly infinite resource of EVs. These EVs have been shown to inhibit liver fibrosis by suppressing TGF-β1/Smad signaling and activating Nrf2/HO-1 pathways [80]. In addition, milk-derived EVs may also display beneficial effects in the progression of liver fibrosis by regulating HSCs [81]. Surprisingly, EVs derived from Schistosoma japonicum have been reported to attenuate liver fibrosis by delivering sja-let-7 to HSCs, providing an updated understanding of parasite–host interactions [82]. Thus, EVs derived from various sources may serve as potential agents for liver fibrosis therapy.

Emerging evidence has demonstrated that EVs can be modified to target HSCs, resulting in improved outcomes for the treatment of liver fibrosis. Notably, the incorporation of a vitamin A derivative into the membrane of unmodified EVs enhances their selective uptake by activated HSCs, thereby increasing therapeutic efficacy in liver fibrosis [83]. HSTP1 is a reliable targeting peptide that specifically binds to activated HSCs, and exosomes modified with HSTP1 exhibit superior targeting capability to these cells, offering a novel strategy for liver fibrosis therapy [84]. Additionally, ADSCs have been engineered to overexpress miRNA-181-5p, which facilitates the selective homing of exosomes to HSCs [85] (Table 1).

**Figure 3:**
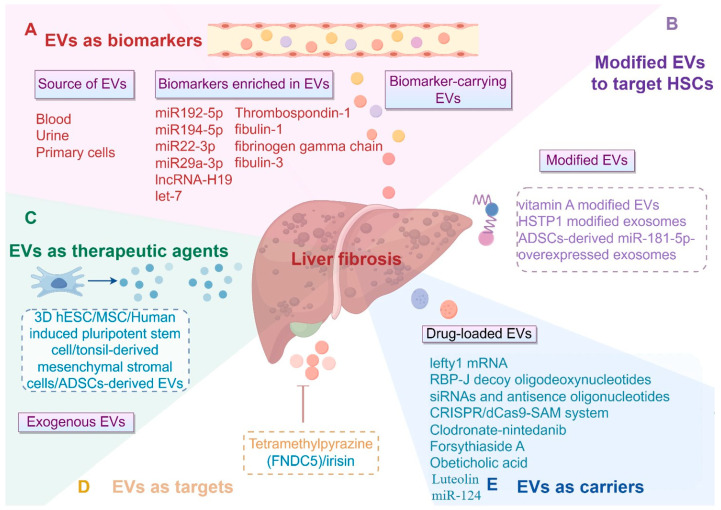
The application of EVs (mainly exosomes and MVs) in liver fibrosis. EVs can serve as biomarkers, targets, therapeutic agents or carriers for liver fibrosis. For example, MSCs represent a source of exosomes that can induce ferroptosis in HSCs by transferring BECN1 and suppress the viability and migration of HSCs through the transfer of miR-618, thereby alleviating fibrosis in mouse liver models. Furthermore, EVs can be engineered to selectively target HSCs by modified with vitamin A, HSTP1, or miR-181-5p. ADSCs, adipose-derived mesenchymal stem cells; hESCs, human embryonic stem cells; MSCs, mesenchymal stem cells.

Recently, EVs have displayed excellent delivery vehicle properties. For example, HSC-derived EVs loading left-right determination factor 1 (lefty1) mRNA have been found to attenuate hepatic fibrosis by blocking the TGF-β/Smad signaling pathway [86]. As shown in the recent study, infusion of exosomes loaded with RBP-J decoy oligodeoxynucleotides (ODNs) could efficiently inhibit Notch signaling in macrophages and ameliorate hepatic fibrosis in mice [87]. In addition, exosomes were modified to carry siRNA, antisense oligonucleotides, or the CRISPR/dCas9 system, which may be one of the effective ways to inhibit liver fibrosis [88,89]. Intriguingly, an exosome–liposome hybrid drug delivery system was utilized to increase the efficacy of clodronate inhibition of KCs and to effectively deliver nintedanib to liver fibroblasts enhance the liver fibrosis therapy [90]. In another study, a CD44-targeting drug delivery system of exosomes encapsulated with Forsythiaside A was developed, which provided a powerful and novel delivery platform with high specificity for liver fibrosis therapy [91]. Obeticholic acid, a farnesoid X receptor (FXR) agonist, when loaded into exosomes, represents a promising approach associated with the inactivation of HSCs, ECM remodeling, and the FXR-Cyp7a1 cascade on CCl_4_-induced liver fibrosis in mice [92]. Luteolin, a plant flavonoid, shows better therapeutic effects when loaded in exosomes for hepatic fibrosis [93]. Exosomes from human Wharton’s jelly mesenchymal stem cells (hWJMSCs) have anti-inflammatory properties and have better anti-inflammatory and anti-fibrotic effects when loaded with miR-124 [94]. Similarly, EVs from hucMSCs loaded with the anti-fibrotic bone morphogenetic protein 7 (BMP7) showed a better therapeutic effect in hepatic fibrosis relative to untreated EVs [95]. Taken together, these studies suggest that exosomes are good vehicles for antifibrotic therapy (Figure 3).

## 4. Migrasomes in Homeostasis and Diseases: Friends or Foes?

Currently, most studies on EVs have focused on exosomes and MVs, but the role of migrasomes in pathophysiological processes of liver fibrosis remains unclear. Migrasomes are newly discovered EVs that form within migrating cells and facilitate intercellular communication. As continuously migrating cells, immune cells have been identified as the primary producers of migrasomes in the body. Notably, it has been reported that neutrophils maintain mitochondria by releasing migrasomes to eliminate damaged mitochondria [96]. Furthermore, Wu et al. observed the biogenesis of migrasomes during neutrophil migration. Migrasomes generated by one neutrophil in mice could be taken up by other neutrophils, and a single migrasome attached to a vessel could sometimes be divided into multiple migrasomes [97]. Recently, studies have shown that neutrophil-derived migrasomes play a crucial role in the coagulation system. These migrasomes are rich in coagulation factors and adhesion molecules, which accumulate at injury sites to initiate platelet activation and promote clot formation [98]. Additionally, macrophage-derived migrasomes containing CD5L have been shown to activate complement-dependent blood–brain barrier damage in a cerebral amyloid angiopathy mouse model [99]. Moreover, migrasomes derived from M2 macrophages have demonstrated the ability to enhance the osteogenic differentiation capacity of MSCs when internalized, suggesting a potential new approach for tissue regeneration [100]. More recently, it has been observed that monocytes deposit migrasomes enriched with pro-angiogenic factors such as VEGFA and CXCL12 to facilitate angiogenesis [101]. Collectively, these studies indicate that immune cell-derived migrasomes may modulate the process of liver fibrosis by regulating the activation of HSCs, the accumulation of extracellular matrix, immune homeostasis, and angiogenesis. Furthermore, migrasomes released by migrating HSCs may also contribute to the onset and progression of liver fibrosis by modulating the immune response [102]. For example, macrophages can influence the activation and proliferation of HSCs through the release of soluble mediators [103], indicating that migrasomes from migrating HSCs could potentially impact the progression of liver fibrosis by modulating macrophage activity.

Recent studies have identified migrasomes as playing a pivotal role in tumor progression. High expression levels of CD151 are closely associated with poor survival outcomes in patients with HCC. Elevated CD151 levels promote the formation of migrasomes, which can enhance the invasiveness and angiogenesis of liver cancer cells, thereby facilitating the progression of HCC [104]. Moreover, pancreatic cancer cells can generate migrasomes that are enriched with immunosuppression-inducing factors. These migrasomes can be taken up by macrophages, leading to the expression of high levels of M2-like markers and the secretion of tumor-promoting factors. Migrasome-induced macrophages play a crucial role in inhibiting T cell proliferation and activation, partially through the action of ARG-1 [105].

**Figure 4:**
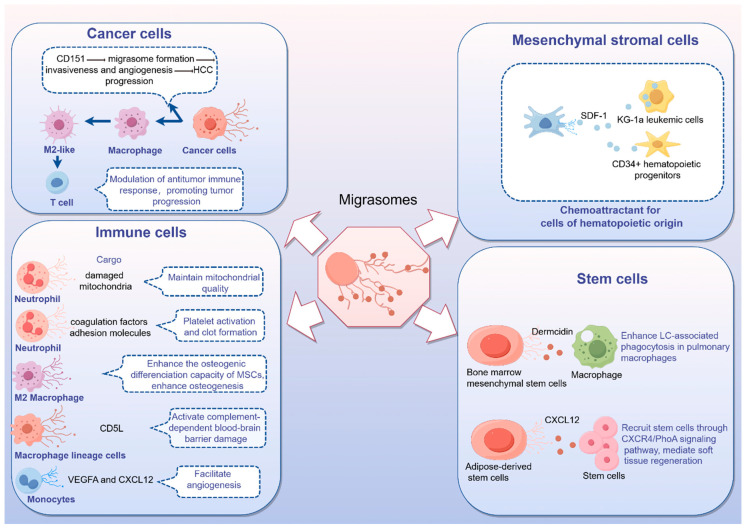
The effect of migrasomes derived from various sources. Migrasomes, a recently identified type of EV generated during cell migration, have been observed in immune cells, cancer cells, mesenchymal stromal cells, and stem cells, and they are playing a crucial role in homeostasis and diseases.

Additionally, mesenchymal stromal cells are capable of generating migrasomes that contain signaling molecules like stromal cell-derived factor 1 (SDF-1), enabling communication with cells of hematopoietic origin [106]. Furthermore, a separate study demonstrated that migrasomes derived from bone marrow mesenchymal stem cells, which contain dermcidin, enhance LC3-associated phagocytosis in pulmonary macrophages [107]. In another study, migrasomes from adipose-derived stem cells are enriched with CXCL12, which plays a key role in recruiting stem cells through the CXCR4/RhoA signaling pathway. This positive feedback loop facilitates soft tissue regeneration (Figure 4). These migrasomes have the potential to restore tissue regeneration by attracting stem cells, demonstrating their promising application in regenerative medicine [108].

**Figure 5:**
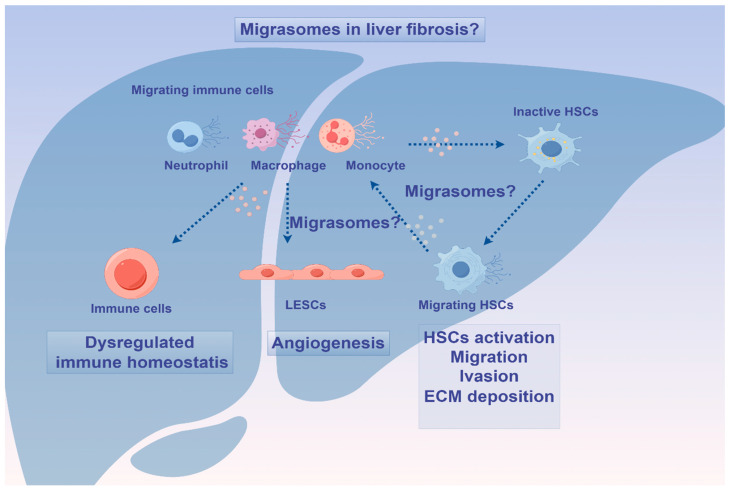
The probable role of migrasomes in pathophysiological processes of liver fibrosis. Immune cell-derived migrasomes may modulate the process of liver fibrosis by regulating the activation of HSCs, accumulation of extracellular matrix, angiogenesis, and immune homeostasis. In turn, migrasomes from migrating HSCs may also be involved in the occurrence and development of liver fibrosis by regulating the immune response. ECM, extracellular matrix; HSCs, hepatic stellate cells; LSECs, liver sinusoidal endothelial cells.

In conclusion, the role of migrasomes derived from different cells in liver fibrosis is complex, and they may affect the activation of HSCs and the repair of liver cells through various ways, thus participating in the occurrence and development of liver fibrosis (Figure 5). In order to better understand the role of migrasomes in liver fibrosis, further research and exploration are needed.

## 5. Migrasomes: Novel Diagnostic and Therapeutical Opportunities?

Migrasomes are capable of transporting a diverse array of cargoes, including nucleic acids, proteins, lipids, enzymes, and metabolites, which provide valuable insights into migratory cell physiology [16,109]. These components have been identified in blood and urine specimens [98,110], underscoring their potential as a promising non-invasive source for liquid biopsy-based disease diagnosis. For instance, the levels of urinary podocyte-derived migrasomes were significantly elevated in kidney disease patients with podocyte injury compared to healthy volunteers. Therefore, the quantification of urinary podocyte-derived migrasomes may represent a promising approach for the early diagnosis of kidney disease associated with podocyte injury [111]. In terms of diagnosis of liver fibrosis, by detecting the number and phenotype of migrasomes in the serum of patients with liver fibrosis, it may be possible to assess the extent and activity of liver fibrosis, which may help in the diagnosis and monitoring of the disease.

Regarding treatment, migrasomes have the potential to be a new target for liver fibrosis treatment. On the one hand, it may be possible to control the process of liver fibrosis by regulating the production and release of migrasomes in specific cells through drug or gene editing techniques. On the other hand, utilizing migrasomes as carriers, it might be possible to transfer anti-hepatic fibrosis drugs or genes into HSCs or other liver cells, thereby enhancing drug efficacy while minimizing side effects. Here, by altering the biomolecules carried by migrasomes, such as reducing pro-fibrotic factors or increasing anti-fibrotic factors, it may have a positive impact on liver fibrosis therapy.

In summary, as a unique type of EVs, migrasomes may play an important role in the diagnosis and treatment of liver fibrosis, providing new ideas and methods for the research and treatment of liver fibrosis. However, the cell migration dependence of the migrasome may affect its preparation. Notably, there are still many challenges and questions regarding the mechanism of action and clinical application of migrasomes in liver fibrosis, which need further research and exploration.

## 6. Clinical Trials of EVs in Liver Fibrosis

Recent clinical trials are investigating the therapeutic potential of MSC-derived exosomes as an alternative to cell therapy for patients with cirrhosis (NCT05871463) (https://clinicaltrials.gov/). Additionally, a study involving 242 patients with cirrhosis demonstrated that circulating levels of hepatocyte MVs can predict patient survival at six months (NCT03837444) [37]. Another study showed that combining hepatocyte-derived biomarkers (keratin-18 and hepatocyte large EVs) with fibrosis testing or the model for end-stage liver disease (MELD) score identifies patients at high risk for liver-related events at 2 years in patients with Child–Pugh class A alcohol-related cirrhosis [112].

## 7. Discussion and Future Challenges of EVs

### 7.1. Advantages and Disadvantages of EV Biomarkers Versus Traditional Techniques

EVs, particularly exosomes, have emerged as promising biomarkers in the field of diagnostics and therapeutics. When comparing EVs as biomarkers to traditional techniques, several advantages and disadvantages must be considered.

#### 7.1.1. Advantages of EV Biomarkers

EVs have been shown to carry a variety of proteins, lipids, and nucleic acids that can indicate specific diseases, offering high sensitivity and specificity in diagnostics. Since EVs can be isolated from various biofluids such as blood, urine, and saliva, they provide a non-invasive approach to disease monitoring and diagnosis. Moreover, EVs can potentially be employed for real-time monitoring of disease progression and therapeutic response, as they are released by cells into the bloodstream and other biofluids.

#### 7.1.2. Disadvantages of EV Biomarkers

Traditional isolation methods for EVs, such as ultracentrifugation, often yield low recovery rates and efficiencies, requiring extended processing times. This limitation can hinder the practical application of EVs as biomarkers. Furthermore, preparing samples for EV isolation can be complex and may involve multiple steps, including pre- and post-processing, which can affect the integrity of the exosomes. Additionally, ensuring the purity of EV preparations can be challenging due to the potential for contamination with other EVs or cellular debris. The lack of standardized protocols for EV isolation and characterization can lead to variability in results across different studies and clinical settings [113]. While commercial kits for exosome isolation are available, their high cost may limit accessibility, particularly in resource-limited settings. Emerging techniques, such as microfluidics, necessitate specialized equipment and expertise, which may not be readily available in all diagnostic laboratories.

In conclusion, although EVs present significant potential as biomarkers due to their unique properties and the extensive information they convey, several challenges regarding their isolation, standardization, and application must be addressed to fully harness their potential in clinical practice. In contrast, traditional biomarker techniques, although more established and accessible, may not offer the same level of sensitivity and specificity that EV-based diagnostics can provide.

### 7.2. Limitations of EV-Based Therapy

One of the primary challenges in EV research is the lack of standardized protocols for the isolation, characterization, and therapeutic application of exosomes. This variability complicates the translation of exosome research into clinical practice and leads to inconsistent results. Furthermore, EV-based therapies face significant limitations with respect to scalability and stability. The production and purification of exosomes can be complex, and maintaining their stability during storage and transportation presents a considerable challenge. Additionally, targeting EVs to specific tissues or organs poses significant difficulties. Off-target effects, limited tissue penetration, and rapid clearance can diminish therapeutic efficacy and result in unintended side effects. While EVs demonstrate improved biocompatibility and reduced immunogenicity compared to synthetic nanoparticles, concerns remain regarding their interactions with the immune system. Changes in the EV surface membrane structure, due to physical loading methods such as extrusion, can alter their immunogenicity profile, making them more recognizable to the immune system and potentially cytotoxic to target cells. The physical forces employed in methods like extrusion can disrupt the EV surface membrane structure, leading to instability or alterations in intrinsic properties. Changes in zeta potential during the loading process can induce cytotoxicity, undermining the potential therapeutic benefits of the loaded cargo. The process of loading therapeutic cargo into EVs can be intricate and may involve multiple steps, potentially affecting the integrity of the exosomes and the therapeutic efficacy of the cargo. Therefore, there is a pressing need for a deeper understanding of EV biology and mechanisms of action, as well as more clinical data to substantiate the safety and efficacy of EV-based therapies.

### 7.3. The Factors Determining the Selection of EVs as a Cargo

The selection of EVs as a cargo delivery system is influenced by a range of factors, including biocompatibility, cargo loading efficiency, stability, cell-to-cell communication, biodegradability, scalability, and regulatory approval. These factors collectively contribute to the potential of EVs as a powerful and versatile tool for therapeutic and diagnostic applications.

The mechanism by which EVs influence liver fibrosis is complex and varied, highlighting their significant potential as therapeutic targets. Future research should prioritize elucidating the specific mechanisms of EVs and developing effective therapeutic strategies. However, utilizing EVs in molecular diagnostics presents challenges, as it necessitates the standardization of EV separation techniques and the characterization of biofluids. Furthermore, establishing unique cargo characteristics for specific stages and types of liver diseases is essential for the application of EVs in molecular diagnostics and personalized medicine in the future. Several unresolved questions remain regarding the use of EVs as therapeutic strategies, including their kinetics, toxicity, off-target effects, and delivery to precise cell types.

## Figures and Tables

**Table 1:** The therapeutic effect of EVs in liver fibrosis derived from various sources.

Sources/Type	Cargos	Target or Outcomes	References
3D-hESCs; exosomes	miR-6766-3p	HSCs; TGFβRII-SMADs, anti-fibrotic	[60]
HucMSCs; exosomes	BECN1	HSCs; xCT/GPX4, HSCs ferroptosis	[61]
MSCs; exosomes	miR-618	HSCs; Smad4, inactivate HSCs	[62]
MSC; exosomes	circDIDO1	HSCs; miR-141-3p/PTEN/AKT	[63]
hBM-MSCs; exosomes	/	HSCs; Wnt/β-catenin, inactivate HSCs	[64]
Placenta MSCs; EVs	miR-378c	HSCs; SKP2, inactivate HSCs	[65]
ADSCs; EVs	miR-150-5p	HSCs; CXCL1, inhibit HSC activation	[66]
Human ADSCs; exosomes	/	PI3K/AKT/mTOR, choline metabolism	[67]
Amnion-derived MSCs; EVs	/	HSCs, Kupffer cells	[68]
MSCs; exosomes	miR-148a	Macrophages; KLF6/STAT3, modulate macrophage phenotype	[69]
HucMSCs; exosomes	/	Hepatocytes; EMT	[70]
Human iPSCs; EVs	miR-92a-3p	HSCs; reduce HSC activation	[72]
Human LSCs; EVs	miR-146a-5p	Anti-fibrotic	[73]
Human LSCs; EVs	/	Anti-fibrotic	[74]
LSCs; exosomes	miR-142-5p	Macrophages; CTSB, regulate macrophage polarization	[75]
T-MSCs; small EVs	miR-486-5p	HSCs; hedgehog signaling	[76]
ASCs; exosomes	/	HSCs; inactivate HSCs, remodel glutamine metabolism	[77]
HUCPVCs; EVs	IGF-I	HSCs, macrophages	[78]
Human UCB; exosomes	/	HSCs; TGF-β/ID1	[79]
HiHep; EVs	/	TGF-β1/Smad, Nrf2/HO-1	[80]
Milk; EVs	/	HSCs; inactivate HSCs	[81]
Schistosoma japonicum; EVs	Sja-let-7	HSCs; Col1α2/TGF-β/Smad	[82]
MSCs; vitamin A-coupled EV	/	activated HSCs; anti-fibrotic	[83]
HucMSCs; HSTP1-exosomes	/	inactivate HSCs	[84]
miR-181-5p-modified ADSCs; exosomes	miR-181-5p	HSCs; autophagy activation	[85]

ADSCs, adipose-derived mesenchymal stem cells; ASCs, adipose-derived stromal cells; EMT, epithelial-to-mesenchymal transition; EVs, extracellular vesicles; hBM-MSCs, human bone mesenchymal stem cells; hESCs, human embryonic stem cells; hiHep, human-induced hepatocytes; HSCs, hepatic stellate cells; hucMSCs, human umbilical cord mesenchymal stem cells; HUCPVCs, human umbilical cord perivascular cells; IGF-I, insulin growth factor like-I; iPSCs, induced pluripotent stem cells; LSCs, liver stem cells; miR, microRNA; MSCs, mesenchymal stem cells; T-MSCs, tonsil-derived mesenchymal stromal cells; UCB, umbilical cord blood.

## Abbreviations

ADSCs, adipose-derived mesenchymal stem cells; ASCs, adipose-derived stromal cells; BMP7, bone morphogenetic protein 7; CCl4, carbon tetrachloride; ECM, extracellular matrix; EMT, epithelial-to-mesenchymal transition; EVs, extracellular vesicles; EXODUS, exosome detection via the ultrafast-isolation system; FFA, free fatty acid; FNDC5, fibronectin type III domain-containing protein 5; FXR, farnesoid X receptor; GLUT1, glucose transporter 1; hBM-MSCs, human bone mesenchymal stem cells; HCC, hepatocellular carcinoma; hESCs, human embryonic stem cells; hiHep, human-induced hepatocytes; HSCs, hepatic stellate cells; hucMSCs, human umbilical cord mesenchymal stem cells; HUCPVCs, human umbilical cord perivascular cells; hWJMSCs, human Wharton’s jelly mesenchymal stem cells; IGF-I, insulin growth factor like-I; interleukin-6, IL-6; iPSCs, induced pluripotent stem cells; KCs, Kupffer cells; Lefty1, left-right determination factor 1; LncRNA, long non-coding RNA; LPS, lipopolysaccharide; LSCs, liver stem cells; LSECs, liver sinusoidal endothelial cells; MASLD, metabolic dysfunction-associated steatotic liver disease; MASP1, mannan-binding lectin serine protease 1; MiR, microRNA; MSCs, mesenchymal stem cells; mtDNA, mitochondrial DNA; MVs, microvesicles; MVBs, multivesicular bodies; NAFLD, non-alcoholic fatty liver disease; ODNs, oligodeoxynucleotides; PDGFα, platelet-derived growth factor receptor alpha; PIP5K1α, phosphatidylinositol-4-phosphate 5-kinase type I α; PKM2, pyruvate kinase M2; SDF-1, stromal cell-derived factor 1; SMS2, sphingomyelin synthase 2; T-MSCs, tonsil-derived mesenchymal stromal cells; UCB, umbilical cord blood.
